# Pharmacogenomics of Drug Metabolizing Enzymes and Transporters: Relevance to Precision Medicine

**DOI:** 10.1016/j.gpb.2016.03.008

**Published:** 2016-10-08

**Authors:** Shabbir Ahmed, Zhan Zhou, Jie Zhou, Shu-Qing Chen

**Affiliations:** 1 Department of Precision Medicine and Biopharmaceutics, College of Pharmaceutical Sciences, Zhejiang University, Hangzhou 310058, China; 2 International Center for Precision Medicine, Zhejiang California International NanoSystems Institute, Hangzhou 310058, China

**Keywords:** Pharmacogenomics, Precision medicine, Genetic polymorphism, Phase-I drug-metabolizing enzymes, Drug transporters

## Abstract

The interindividual genetic variations in drug metabolizing enzymes and transporters influence the efficacy and toxicity of numerous drugs. As a fundamental element in **precision medicine**, **pharmacogenomics**, the study of responses of individuals to medication based on their genomic information, enables the evaluation of some specific genetic variants responsible for an individual’s particular drug response. In this article, we review the contributions of **genetic polymorphisms** to major individual variations in drug pharmacotherapy, focusing specifically on the **pharmacogenomics** of phase-I drug metabolizing enzymes and transporters. Substantial frequency differences in key variants of drug metabolizing enzymes and transporters, as well as their possible functional consequences, have also been discussed across geographic regions. The current effort illustrates the common presence of variability in drug responses among individuals and across all geographic regions. This information will aid health-care professionals in prescribing the most appropriate treatment aimed at achieving the best possible beneficial outcomes while avoiding unwanted effects for a particular patient.

## Introduction

Pharmacogenomics is the understanding of how individuals differ in their response to drug therapy and the mechanisms underlying variable drug response by utilizing genomics, proteomics, transcriptomics, and metabolomics based knowledge. Every individual has a different genetic makeup, which influences the risk of developing diseases as well as responses to drugs and environmental factors [1]. Genomic differences between individuals are present approximately every 300–1000 nucleotides with over 14 million single nucleotide polymorphisms (SNPs) distributed throughout the entire human genome [2]. Therefore, identification of DNA variants that most significantly contribute to the population variations in each trait is one of the fundamental objectives of genetics [3]. The understanding of variations in interindividual drug response behaviors has been greatly improved owing to the rapid developments in pharmacogenomics over the last few years. Each individual in a large patient population responds differently, which possibly explains why a treatment that has been proven efficacious in some patients often fails to elicit adequate responses in others. Moreover, such treatment failure in the affected patients may cause some serious side effects or even lead to death, which is inductive of individual variability in drug safety and efficacy. The causative factors for variations in drug response are complex and multifold with direct or indirect consequences. Among them, stably-inherited genetic factors are the major variables [4], whereas others include environmental factors like chemicals and radiation exposure, lifestyle factors like drinking, smoking and exercise, and physiological factors like age, sex, liver and kidney function, pregnancy, and starvation [5]. It is evident from previous studies that population variability in drug response is often larger than intrapatient variability (within the same individual at different time points) [6].

Drug response of individual patients is primarily determined by the pharmacokinetic and pharmacodynamic properties of prescribed drugs, which is directly or indirectly affected by polymorphisms in drug metabolizing enzymes and transporters. Different populations have varied allele frequencies in genes of both drug metabolizing enzymes and transporters. For precision medicine, the molecular and clinical information is integrated in order to understand the biological basis of disease and develop medications with better outcomes for patients [7]. Therefore, precision medicine will help to improve the selection of disease targets and lead to the identification of patient populations that exhibit better clinical result at normal doses [8].

## Variations in drug response

It is well known that individuals vary significantly in their clinical responses to administered drugs and the outcomes, which can be inherited or acquired, are always patient-specific [9]. Such interindividual variation is often a challenge to optimizing a dosage regimen because most drugs are effective in only 25%–60% of patients [10]. Many patients are unable to fully respond and benefit from the first recommended drug treatment. For example, an average of 38%, 40%, 43%, 50%, and 75% of patients who have depression, asthma, diabetes, arthritis, and cancer, respectively, show no response to initial treatments [11].

Different patients can respond differently to the same drug and dose. Sometimes, the effective drug dose for a particular patient may prove lethal to or result in therapeutic failure in others (too low drug concentrations at normal doses), leading to serious adverse effects or no effects at all. Continuous drug monitoring is recommended when prescribing drugs with known serious side effects and narrow therapeutic indexes to avoid unexpected and undesirable outcomes [12]. The situation can worsen if the patient takes other drugs and has other existing disease conditions due to possible drug–drug and drug–disease interactions [13]. For example, the daily warfarin dose varies by up to 20- to 30-fold between patients in many disease conditions where it is recommended for the treatment of embolism and thrombosis [13]. Similar observation has also been reported for dose-dependent individual variations in drug response to simvastatin, an inhibitor of 3-hydroxy-3-methyl-glutaryl-coenzyme A reductase (HMGCR) [14].

The recommended daily maximum dose of simvastatin for the management of blood cholesterol levels is 40 mg. In a cohort study of 156 patients, 95% of them showed reduced levels of low-density lipoprotein (LDL) cholesterol, whereas the remaining 5% exhibited no reduction was observed for the remaining 5% of the patients, even at doses as high as 160 mg/day of simvastatin [15]. It is suggested that the genetic polymorphisms in genes encoding ATP-binding cassette sub-family G member 2 (ABCG2) and HMGCR contribute to the interindividual difference in a dose-dependent manner [14], [16].

## Contributing factors in interindividual drug responses

Individual-specific response to medication can be attributed to many multifold and complex factors including the unique genetic makeup (mutations such as SNPs, gene deletions, and duplications). These genetic factors, as well as physiological conditions (age, gender, body size, and ethnicity); environmental influences (exposure to toxins, diet, and smoking); and pathological factors (liver and renal function, diabetes, and obesity) can work alone or in combination to influence drug responses [17]. According to the hypothesis of Tang et al. [18], various genetic factors contribute approximately 20%–95% to determining the interindividual variability in drug responses. Furthermore, individual variations in responses related to genetic factors are often permanent, while those influenced by other factors are mostly transient [6]. In support of inheritance being a major determinant of drug response, Vesell et al. [19] found relatively higher population variability of a drug response among all the individuals in a population than the intrapatient variability at different times.

## Determinants of interindividual drug responses

Disease conditions of individuals used to be diagnosed based on signs and symptoms, which may be indicative of several different diseases or somewhat related to the family history. In the past, clinicians could only attempt to cure or treat disease upon its onset [20]. Currently, more specific and precise diagnostic approaches have been developed to examine genes and the genetic variants known to be associated with altered interindividual drug response or specific diseased conditions. Success of the Human Genome Project (HGP) has contributed considerably in this context. Pharmacogenomics enables scientists to assess specific genetic variants that may be responsible for an individual’s particular drug response by identifying the particular genetic loci involved [21]. Whole-genome SNP profiling, haplotyping, multigene analysis, and gene expression studies using biochips or microarrays [22], [23] are recently used to study individual responses to drugs at various levels and could facilitate drug discovery and development [24].

**Figure 1:**
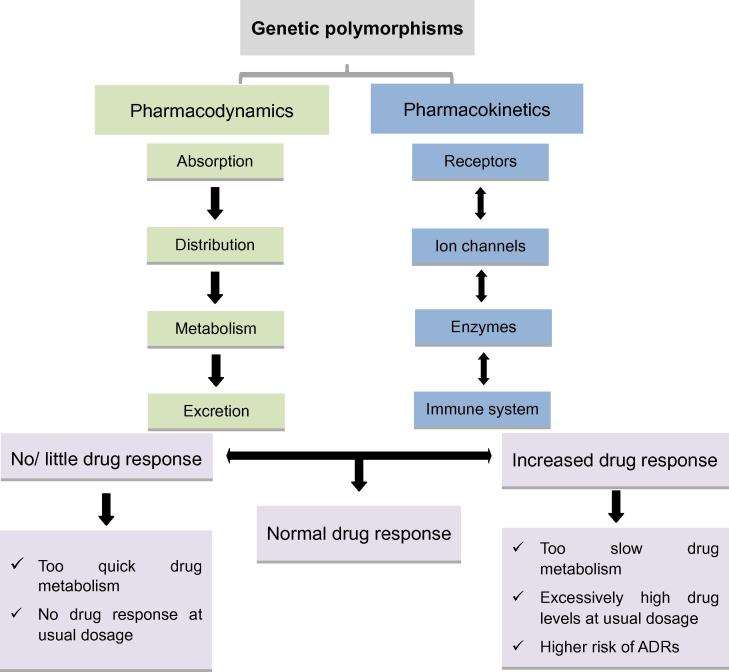
Effect of genetic polymorphisms on individuals’ drug response Pharmacokinetics and pharmacodynamics are main determinants of interindividual differences in drug responses. Genetic polymorphism in genes related to these processes may result in mild to severe variations in drug responses. ADRs, adverse drug reactions.

Genetic polymorphisms may influence a drug’s effect by altering its pharmacokinetics, pharmacodynamics, or both (Figure 1), which are two major determinants conferring the interindividual differences in drug responses. Pharmacokinetics deals with how much of a drug is required to reach its target site in the body, while pharmacodynamics deals with how well the targets such as receptors, ion channels, and enzymes respond to various drugs [25], [26]. Genetic polymorphisms in drug transporters and phase-1 drug-metabolizing enzymes can alter the pharmacokinetic and pharmacokinetic properties of the administered drugs, their metabolites or both at the target site, resulting in variability in drug responses. Theoretically, variations at even a single base (SNPs) or sets of closely-related SNPs (haplotypes) in genes involved in the pharmacokinetic and pharmacodynamic pathways at any stage could affect the overall drug response of an individual [27], [28].

Mutations in the gene coding regions could cause alterations in gene expression or protein structure, leading to variations in protein quantity and quality. In the case of enzymes, such mutations affect both the protein function and the rate and kinetic constants. Changes in drug-receptor or drug–enzyme interactions due to structural alterations of enzymes or receptors could also result in variations in drug responses [6]. Polymorphisms in genes responsible for drug transport can affect pharmacokinetic properties of an administered drug and ultimately its plasma concentration as well as concentrations in the target tissues. In addition, altered drug response could also be attributed to reduced repairing capability for mutations triggered by alkylating agents due to malfunctioning of DNA repair enzymes [29]. Such protective effect could be affected by genetic polymorphisms causing altered protein structure or reduced expression in enzymes responsible for glutathione biosynthesis [2].

Twin studies have provided evidence supporting the contribution of genetic factors to individuals’ varied drug responses. For instance, in the late 1950s, it was found that dizygotic twins exhibited more metabolic variability than did monozygotic twins for isoniazid metabolism [30]. Subsequent investigations of halothane, antipyrine, and phenytoin metabolism in twins revealed the major influence of genetic factors and exposure to disease-favoring environment [31], [32] .

## Influence of polymorphisms in genes encoding phase-I drug metabolizing enzymes

### Cytochrome P450 2D6

Cytochrome P450 (CYP), which represents a large and diverse group of heme-containing enzyme superfamily, is involved in oxidative metabolism of structurally-diverse molecules like drugs, chemical, and fatty acids. The genetic polymorphism in the genes encoding CYP members was firstly reported for *CYP2D6*. The highly polymorphic *CYP2D6* gene is located on the chromosome 22q13.1, consisting of nine exons and eight introns (GenBank accession No. NM 000106.5) [33], [34]. More than 100 *CYP2D6* genetic variants have been described (http://www.cypalleles.ki.se/cyp2d6.htm) to date, resulting from point mutations, duplication, insertions or deletions of single or multiple nucleotides, and even whole-gene deletion. Individuals carrying different CYP2D6 allelic variants have been classified as poor metabolizers (PMs), intermediate metabolizers (IMs), extensive metabolizers (EMs), and ultrarapid metabolizers (UMs) according to the metabolic nature of the drugs and degree of involvement in drug metabolism of these variants [35]. Although constituting only 2%–4% of the total amount of CYPs in the liver, CYP2D6 actively metabolizes approximately 20%–25% of all administered drugs [36]. The drugs metabolized by CYP2D6 include tricyclic antidepressants, serotonin reuptake inhibitors, antiarrhythmics, neuroleptics, and β-blockers [35].

The extensive presence of polymorphism in the *CYP2D6* gene significantly affects phenotypic drug responses. Up to a 10-fold difference in the required dose was observed in order to achieve the same plasma concentration in different individuals [37]. Dextromethorphan, debrisoquine, bufuralol and sparteine are the probe drugs used for *in vivo* CYP2D6 phenotyping. According to the probe substrate metabolic capabilities among the sampled individuals in a population, patients can be categorized into the following four phenotypic groups: poor, intermediate, extensive, and ultra-rapid metabolizers (PMs, IMs, EMs, and UMs), respectively [38]. The interindividual phenotypic variations depend on the metabolic properties of the *CYP2D6* allelic variants (Table 1). Simultaneous presence of two null (non-functional) alleles in an individual [39] confers a PM phenotype, whereas individuals with two normally-functioning alleles [40] present with the EM phenotype. In addition, co-existence of a null allele with another allele associated with reduced function [41], [42] gives rise to an IM phenotype, whereas presence of extra *CYP2D6* gene copies with normal activity confers the UM phenotype. According to the CYP2D6 phenotype, the Caucasian population comprises approximately 5%–10% PMs, 10%–17% IMs, 70%–80% EMs, and 3%–5% UMs [39]. The percentages of PMs, IMs, EMs, and UMs differs among different ethnicities due to the significant variability in the *CYP2D6* allele distribution (Table S1 and Table S2).

Individuals with the UM phenotype can metabolize the administered CYP2D6 substrates in much shorter time than individuals with the IM or PM phenotypes [43]. This leads to very low plasma drug levels with potential loss of drug efficacy. Therefore, higher drug doses would be required to attain effective drug concentrations, which could be fatal when dealing with drugs with narrow therapeutic indexes. Notably, a large number (approximately 10%–30%) of Saudi Arabians and Ethiopians have been reported to have the CYP2D6*2XN allele [44], [45]. On the other hand, there is an opposite situation for the individuals with the CYP2D6*3, *4, *5, and *6 alleles (PM phenotype). These allelic variants lead to inactive CYP2D6 enzymes [46], [47], [48], [49], [50]. As a result, the affected individuals exhibit high plasma drug levels with increased risks of drug-related side effects and therefore reduced drug dose should be administered [51]. The allelic frequencies with clinical consequences of CYP2D6*3 (3.3% in Sardinians), CYP2D6*4 (23%–33% in Polish and Faroese populations), CYP2D6*5 (5.9%–6.2% in Spaniards and African Americans), and CYP2D6*6 (1.9%–3.3% in Faroese and Italians) were also calculated in diverse populations (Table S2).

The prodrug tamoxifen is a selective estrogen receptor (ER) modulator used to treat ER-positive breast cancer patients [52]. Tamoxifen is actively catalyzed to endoxifen and 4-hydroxytamoxifen by various CYPs with CYP2D6 acting as the rate-limiting enzyme [53]. Plasma level of endoxifen in UM patients is usually higher than that in PM and IM patients due to the presence of multiple functional *CYP2D6* copies [53]. The presence of *CYP2D6* null alleles in high frequencies commonly contributes to the CYP2D6 PM phenotype in individuals, as is the case with the CYP2D6*4 (33%) in the Faroese population [47]. In tamoxifen-treated surgically resected ER-positive breast cancer patients, a much lower (0) prevalence of moderate to severe hot flashes, together with a higher risk of disease relapse, was reported in women with the CYP2D6*4/*4 genotype than in patients with one or no CYP2D6*4 alleles (20%) [54]. Codeine is a commonly prescribed analgesic, which is converted to its active metabolite morphine and acts at mu-opioid receptors to induce analgesia. The affinity of morphine to mu-opioid receptors is 200-fold stronger than that of codeine [55]. Interestingly, conversion from codeine to morphine is also catalyzed by CYP2D6, which has been proven as the key enzyme responsible for the analgesic effect of codeine. The CYP2D6 phenotype is therefore a critical determinant in opioid analgesia. According to McLellan et al. [45], subjects with the PM phenotype can only convert 10% of a codeine dose to morphine while approximately 40% and 51% conversion occurs in EMs and UMs, respectively. Thus, in individuals with null allelic variants of *CYP2D6*, codeine is not recommended as an analgesic because of the minimal enzymatic conversion from codeine to morphine. Conversely, a higher risk of morphine toxicity may occur in patients with the UM phenotype owing to the rapid conversion of codeine to morphine. The situation would be more devastating in UMs who are lactating mothers because the normal codeine dose can translate into fatal morphine concentrations into the breast milk [56]. In 2006, a case of a 13-day newborn death was reported when the infant’s mother was placed on the codeine therapy after delivery for pain management of episiotomy [57]. There are also other cases reporting that the routinely recommended codeine doses produced lethal adverse effects in UM patients [56], [58], [59]. The *CYP2D6* allelic variants *10, *17, and *41 exhibit normal catalytic activity but are sometimes associated with intermediate to low metabolic activities [60]. In the Chinese population, the CYP2D6*10 allele has been found more common than other alleles (allelic frequency of up to 65%) and it causes a greatly decreased (but not deficient) enzyme activity [61].

### CYP2C9

CYP2C9 is another important member of the CYP superfamily. The gene coding for CYP2C9 is located on chromosome 10q24.2, and spans more than 55 kb in length. CYP2C9 constitutes approximately 18% of the total CYP protein in the human liver microsomes [62]. CYP2C9 metabolizes approximately 25% of clinically-administered drugs including anti-inflammatory agents such as flurbiprofen, hypoglycemic agents such as glipizide and tolbutamide, the anticoagulant S-warfarin, and the anticonvulsant phenytoin [63], [64]. More than 60 variant alleles have been identified for the *CYP2C9* gene (http://www.cypalleles.ki.se/cyp2c9.htm). Among them, CYP2C9*2 (R144C) and CYP2C9*3 (I359L) are the most common variants associated with highly-reduced CYP2C9 enzymatic activities in comparison with the wild-type allele (CYP2C9*1) [65].

The CYP2C9*2 variant results in a markedly decreased enzyme activity due to higher km value and lower intrinsic clearance of drugs like S-warfarin [16]. The CYP2C9*2 allelic variant has been reported with up to 25% allelic frequencies in the Iranian population [66]. However, frequencies of heterozygous CYP2C9*1/*2, homozygous CYP2C9*2 or CYP2C9*3 carriers were lower (0.1%–1%) in the Chinese and Japanese populations compared with those in Caucasians and Iranians. Caucasians have approximately 1% CYP2C9*2 and 0.4% CYP2C9*3 homozygotes, respectively [67]. Furthermore, approximately one-third of the Turkish population has either the *1*2 or the *1*3 genotype, while more than 2% have the *2*2, *2*3, and *3*3 genotypes [68]. In the Iranian and Pakistani populations, the prevalence of CYP2C9*2 and CYP2C9*3 is greater than that in the other studied populations [66]. On the other hand, Chinese, Vietnamese, Korean, Bolivian, and Malaysian populations have a CYP2C9*1 allelic frequency variant of >90%, whereas allelic CYP2C9*2 variant was not detected in the Korean, Chinese, and Vietnamese populations but occurs 1% in the Japanese. Furthermore, no individuals from the South African and Zimbabwean populations have been reported to carry the CYP2C9*2 allele (Table S1).

The interindividual and interethnic variations in the *CYP2C9* polymorphisms are clinically significant especially in the patients on anticoagulation therapy with warfarin. Warfarin is one of the most widely-prescribed oral anticoagulants [13]. Clinically-available warfarin is a racemic mixture of the R and S enantiomers, with the S-isomer exhibiting an approximately 5-fold higher anticoagulant potency than the R-isomer [69]. Inactivation of the active S-warfarin is almost exclusively mediated by CYP2C9. Patients with high allele frequencies of the *CYP2C9* wild-type or CYP2C9*1 excrete the S-warfarin normally from the body. In contrast, PMs who have high allelic frequencies of the CYP2C9*2, CYP2C9*3, or both have impaired S-warfarin-metabolizing capabilities and, therefore, require lower drug doses to attain therapeutic responses [70], [71], [72], [73]. Thus, PMs have higher risks of internal bleeding than individuals with higher CYP2C9*1 allelic frequencies during warfarin therapy [69], [72]. Although polymorphisms in genes encoding blood-clotting factors also contribute to the bleeding risk and initial warfarin dose adjustment requirements, *CYP2C9* gene polymorphisms always exert greater influence [74].

Both CYP2C9 and CYP2C19 are involved in microsomal hydroxylation of phenytoin to its R and S enantiomers [75]. Therefore, *CYP2C9* genotype is an important determinant in *in vivo* phenytoin metabolic studies. Due to the narrow therapeutic range of phenytoin, even minimal variations in CYP2C9 activity can be clinically important [76]. In a study on healthy Turkish individuals with already known CYP2C9 genotypes, Aynacioglu et al. [68] reported that subjects with CYP2C9*1/*2, CYP2C9*1/*3, and CYP2C9*2/*2 genotypes had significantly higher phenytoin serum concentrations and lower levels of 5-(4-hydroxyphenyl)-5-phenylhydantoin (phenytoin metabolite) than those with the CYP2C9*1/*1 genotype. Multiple studies have also shown that the CYP2C9*3/*3 genotype is associated with reduced metabolisms and altered pharmacokinetic properties of substrates such as phenytoin, warfarin, losartan, and tolbutamide [77], [78], [79], [80].

### CYP2C19

The polymorphic *CYP2C19*, which is located on the chromosome 10q24 encodes another CYP family member. CYP2C19 can metabolize numerous routinely-administered drugs such as anxiolytics (diazepam), proton pump inhibitors (omeprazole), anticonvulsants (S-mephenytoin), and antimalarial biguanides [35], [81], [82], [83]. Up to now, more than 35 *CYP2C19* variants and approximately 2000 SNPs have been identified (http://www.cypalleles.ki.se/cyp2c19.htm), with continuous increase in SNP numbers reported. Among them, CYP2C19*2 and CYP2C19*3 are the most common variants that have been studied extensively. Both of them are null variants and patients carrying these variants are therefore categorized as PMs. CYP2C19*2 is the most common allelic variant caused by a single nucleotide alteration in exon 5 (G > A), resulting in an abnormal splicing site and conferring reduced enzymatic activities of CYP2C19 [83], [84].

The CYP2C19*2 variant is found at a high allelic frequency (30%) in South Indians, but occurs with the lowest frequency (2.9%) in the Faroeses. In contrast, CYP2C19*3 is found at higher allelic frequencies in the Japanese (approximately 13%) but lower (0) among the Italians, South Africans, Greeks, European-Americans, and other populations (Tables S1 and S2). Approximately 15%–25% of the Korean, Japanese, and Chinese populations have been reported as PMs of the anticonvulsant drug S-mephenytoin [85], [86], [87]. The activity of omeprazole, a drug recommended for treating peptic ulcers and gastroesophageal reflux diseases, was found to be highly patient *CYP2C19* genotypes dependent [88]. Furuta et al. [89] found that after a single dose (20 mg) of omeprazole [90], the observed intragastric pH values were 4.5, 3.3, and 2.1 for PMs, heterozygous EMs, and EMs individuals, respectively. In another study, Schwab et al. [91] reported lower serum concentrations of lansoprazole, a proton pump inhibitor, and lower rates of *Helicobacter pylori* eradication in Caucasian EM patients following a standard dose of lansoprazole. The individuals with the PM phenotype of CYP2C19 required lower doses of the proton pump inhibitor lansoprazole for beneficial therapy than that required by the patients with the EM phenotype of CYP2C19 [92]. Both CYP2C19*2 and CYP2C19*3 variant alleles of *CYP2C19* are associated with inactive enzyme production, which is evident from the various population studies summarized in Table S1. Some drugs strongly affected by *CYP2C19* genotypes, and their labels contain pharmacogenomic information are summarized in Table 2.

### CYP3A4 and CYP3A5

More than 50% of clinically-administered drugs are metabolized by CYP3A4, which is the most abundant CYP enzyme in the liver [93]. Therefore, polymorphisms in *CYP3A4* are of great concern in the study of interindividual altered drug metabolisms and related ADRs [94]. More than 26 *CYP3A4* variants have been identified (http://www.cypalleles.ki.se/cyp3a4.htm) and most of these variants are responsible for varied enzyme activities ranging from modest to highly reduced catalytic efficiencies among the affected individuals [35]. Comparatively, high frequencies of allelic variants of the *CYP3A4* gene (CYP3A4*2 and CYP3A4*3) were observed in Caucasian whereas high frequencies of allelic variant CYP3A4**18 were observed in Chinese people (Table S1). The clinical consequences of different allelic variants of *CYP3A4* are still undefined for many substrates of CYP3A4. Considering the relatively low frequencies, only small changes in the enzyme activity have been caused by CYP3A4*16 and CYP3A4*18 variants [95].

**Figure 2:**
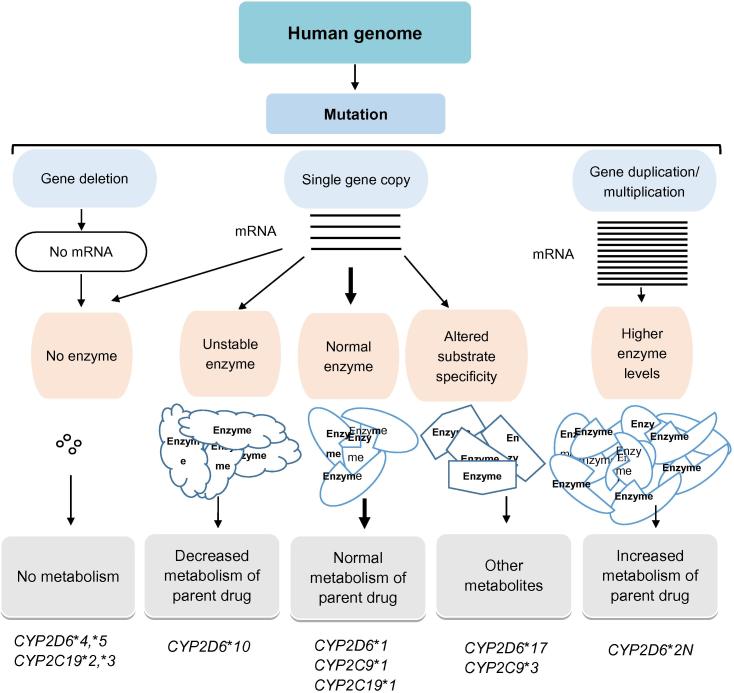
An overview of important consequences of genetic polymorphisms in the CYPs Overview of the effect of genetic polymorphisms on some human cytochrome P450 variant alleles and molecular mechanisms leading to altered drug metabolism.

CYP3A5 is one of the factors that contribute to the complexity of CYP3A4. With few exceptions, CYP3A5 can metabolize most drugs that are substrates of and metabolized by CYP3A4. Although slower in most cases [96], the metabolic activity of CYP3A5 is equal [97] to or even faster than that of CYP3A4 in some cases [98]. *In vivo* studies revealed that the metabolic rates for the drug that are metabolized by both CYP3A4 and CYP3A5 are the sum of the activities of both enzymes. Functionally active variants of *CYP3A5* are expressed in half of the African population and one-fourth of Caucasians [99]. This may partially explain why human studies of the *CYP3A4* allelic variants do not agree with its clinical effects [100]. An overview of the important consequences of gene mutations of the CYPs is illustrated in Figure 2.

CYP oxidoreductase (CYPOR) is the catalytic partner and compulsory element to all CYP-mediated metabolisms. The interaction between the CYP and CYPOR is essential for the metabolic activities of CYPs [14]. CYPOR is required for electron transfer from NADPH to CYP via its FAD and FMN domains, which is crucial for CYP catalytic activities [101], [102]. Therefore, *CYPOR* allele variants like POR*5, POR*13 and POR*27 can indirectly alter the functional consequences of CYPs [103], [104], [105]. For example, in POR*27 variant, L577P mutation located in the NADPH-binding domain of CYPOR [102] leads to decreased CYPOR activity, due to changed helix and disrupted NADPH interaction [105], whereas POR*5 (A287P) is associated with impaired ability to accept electrons from NADPH [106]. Additionally, POR*13 (Q153R) variant leads to severely-impaired steroid biosynthesis in Antley–Bixler skeletal malformation syndrome (ABS) [107]. Until now, more than 50 different variants of the human *CYPOR* genes have been described (http://www.cypalleles.ki.se/por.htm).

## Effect of polymorphisms in genes encoding drug transporters

A drug could produce a beneficial or toxic effect in a particular patient. The nature and extent of the resulting effect is largely dependent on the absorption, distribution, and excretion rates of the drug. Drug transporters primarily control the movement of all drugs and their active or inactive metabolites into or out of cells. Therefore, polymorphisms of drug transporter genes can modify the absorption, distribution, and excretion rates, and ultimately safety and efficacy of the administered drugs. The ABC and solute-carrier (SLC) transporters are two superfamilies of transport proteins are ubiquitous membrane-bound transport proteins that are involved in the absorption, distribution, and elimination of drugs [92].

**Figure 3:**
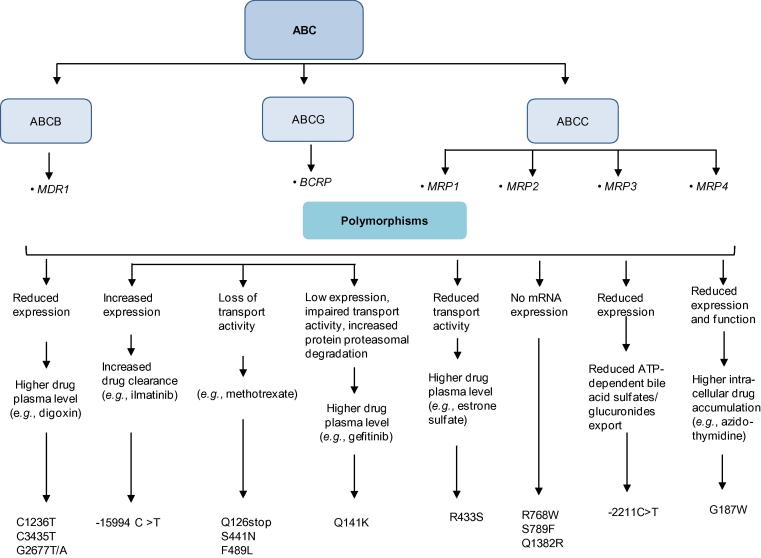
The influence of genetic polymorphisms of ABC transporters on the drug transport activities The diagram depicts the influence of genetic polymorphisms on the drug transport activities of ABC transporters. ABC transporter, ATP-binding cassette transporter; MDR1, multidrug resistance protein 1; BCRP, breast cancer resistance protein; MRP, multidrug resistance-associated protein.

**Figure 4:**
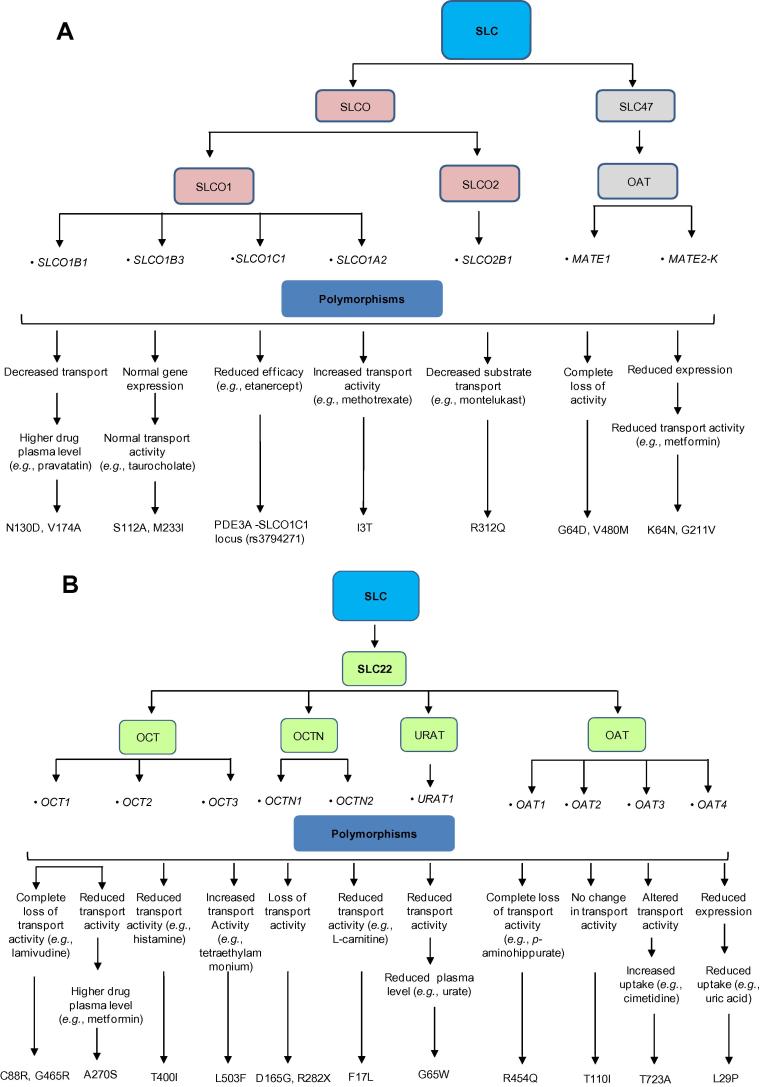
Modification of drug transport activities of SLC transporters by genetic polymorphisms The diagram depicts the influence of genetic polymorphisms on the transport activities of different allele variants of SLC transporters including SLCO and SLC47 (**A**) as well as SLC22 (**B**). SLC, solute carrier; SLCO, solute carrier organic anion; OCT, organic cation transporter; OCTN, organic cation transporter novel; OAT, organic anion transporter; MATE1, multidrug and toxin extrusion protein 1; URAT, urate transporter.

ABC transporters often transport drugs and other substances against the concentration gradient using ATP as an energy source [108]. In ABC transporter superfamily of drug transporters, 49 genes have been identified, which are divided into seven subfamilies from *ABCA* to *ABCG* (http://nutrigene.4t.com/humanabc.htm). The impact of some important polymorphisms on the drug transport activities of various ABC transporters is summarized in Figure 3. In addition, approximately 360 genes have been identified in the SLC superfamily and are classified into 46 subfamilies (http://www.bioparadigms.org/slc/menu.asp). Among them, members of the organic anion transporter (OAT), organic anion transporting polypeptides (OATP), and organic cation transporter (OCT) subfamilies are of particular significance in drug disposition [109]. In addition, polymorphisms in genes encoding SLCO, SLC22, and SLC47 family members within the SLC superfamily have key roles in modulating drug transport activities of the corresponding transporters (Figure 4).

### ABCB1

The *ABCB1* gene, also known as the multidrug resistance 1 (*MDR1*), encodes a P-glycoprotein (Pgp), which is involved in the cellular efflux of numerous chemotherapeutic agents, physiological metabolites, and carcinogens [110]. *ABCB1* is highly polymorphic with allelic variants found in varied frequencies in different populations (Table S3). *ABCB1* polymorphisms were identified firstly by Kioka et al. [111] in different cancer cell lines in 1989 and subsequently by Hoffmeyer et al. [112] and other researchers [113], [114], [115], [116]. As an efflux transporter, ABCB1 is detected on the surface of epithelial cells, preventing intestinal absorption, protecting fetus and brain from xenobiotic exposure and facilitating renal and hepatobiliary excretions [117]. Interestingly, overexpression of the *ABCB1* gene in cancer cells induced resistance to chemotherapeutic agents [110].

Distribution of some allelic variants appears to be ethnicity-dependent. For instance, SNP 3435C > T occurs at high frequencies (60%–72%) in Asians but low (34%–42%) in Caucasians. The substrate-dependent effects of Pgp on pharmacokinetic and pharmacodynamics properties remain obscure due to controversial studies on digoxin disposition. For example, for patients with a mutant allele (3435C > T) that were administered a single oral dose of digoxin, Sakaeda et al. [118] reported lower serum concentrations of digoxin, whereas higher plasma digoxin levels were observed by Verstuyft and her colleagues [119]. The haplotype 1236C > T/2677G > T/3435C > T was detected with high frequency (up to 56%) in Asians [120]. Kimchi-Sarfaty et al. [121] found that patients carrying this haplotype exhibited normal transporter properties although the transporter inhibition by small modulators was affected. The conflicting results of these studies could be indicative of additional polymorphisms yet-to-be-identified other than the studied mutations or might reflect the complex disposition pathways of the substrate drugs in the studied subjects. For example, cyclosporine, a CYP3A4 substrate that is a widely-used immunosuppressant in patients with liver, kidney, or heart transplants, is also transported by ABCB1 [122]. Similarly, fexofenadine and digoxin can be simultaneously transported by OATP and ABCB1. Letourneau et al. [123] studied the transport activity of ABCB1 with R230Q, R633Q, R1056Q, R723Q, T73I, S1512L, S92F, T117M, A989T, or C1047S nonsynonymous SNPs by using different substrates (methotrexate, leukotriene C-4, and estradiol-17-β-glucuronide). However, they failed to find any significant effect of the aforementioned variants on either gene expression levels or transport functions. Conversely, a 50% reduction in transport activity was observed in the A989T variant [124]. Compared to Asians and Caucasians, the 3435C > T allele occurs lowly in Africans, and it has been proposed that this low frequency of the *MDR1 3435T* allele might be associated with the reduced incidence of renal carcinoma in African populations [124]. On the other hand, the *MDR1 3435C* allele might have a protective role in parkinsonism patients with a known history of pesticide exposure [125].

### ABCC1 and ABCC2

As the important ABC members, both ABCC1 and ABCC2 are involved in the transport and excretion of several chemotherapeutic agents, toxicants, and organic anion molecules [128]. Glutathione cotransporter is essential for both of them to transport some substrates such as estrone sulfate [126]. In non-Hodgkin lymphoma patients treated with doxorubicin, significant associations between the G671V variant and a V188E-C1515Y haplotype of *ABCC2* and G671V variant with 28% allelic frequency in Caucasians have been reported [127], [128]. V417I is another widely distributed variant in *ABCC2* (Asians 13%–19%, Africans 14%, and Caucasians 22%–26%) that has been extensively studied for its role in drug resistance development in cancer and human immunodeficiency virus type 1 (HIV-1)-infected patients [129], [130], [131].

### ABCG2

Similar to ABCC1, ABCG2 was first discovered in multidrug-resistant cell lines [132], which is also known as the breast cancer resistance protein (BCRP), mitoxantrone resistance protein (MXR) or placenta-specific ABC protein (ABCP) [133]. ABCG2, which is expressed in the epithelial cells of the small intestine, lung, kidney, sweat glands, colon, and placenta, is essential for intestinal absorption and biliary excretion of drugs and their metabolites and xenobiotic [134]. More than 80 polymorphisms of the *ABCG2* gene have been identified [135]. Among them, SNP C421A in the variant (p.Q141K) has been found to be associated with the reduced expression and altered substrate specificity ABCG2 [136].

The C421A is widely distributed in many ethnicities with frequencies of 27%–35% in Asians, 9%–14% in Caucasians, and 1%–5% in Africans (Table S3). Gefitinib, the inhibitors of epidermal growth factor receptor (EGFR) tyrosine kinase, are substrates of ABCG2. In cancer patients who were treated with gefitinib, presence of C421A was related to increased drug accumulation and higher prevalence of drug-induced grade 1 or 2 diarrhea [137], [138], when compared to patients with wild type allele. In another study, Sparreboom et al. [139] reported a 300% elevation in plasma levels of the anticancer drug diflomotecan in individuals with the heterozygous C421A genotype when the drug was administered intravenously [139]. Presence of C421A also affects the pharmacokinetic and therapeutic effects of rosuvastatin in Chinese and Caucasians. Tomlinson et al. [16] reported the significant influence of C421A in reducing LDL cholesterol levels in a gene- and dose-dependent manner in Chinese patients with hypercholesterolemia [16]. Therefore, a systemic analysis of polymorphisms of *ABC* transporters would be essential to enhance the understanding of the genetic impact on pharmacotherapy.

### OATPs

OATPs are a large family of membrane-bound influx transporters that are responsible for the cellular uptake of a wide range of endogenous and exogenous substances including bile salts, hormones, and clinically administered drugs such as antibiotics, cardiac glycosides, and anticancer agents [140]. There are 11 human OATP transporters, among which OATP1A2, OATP1B1, OATP1B3, OATP2B1, and OATPC are involved in drug pharmacokinetics [138]. In particular, the OATPC*5 and OATPC*9 allelic variants are associated with a reduced uptake of OATPC substrates such as estrone sulfate and estradiol-17-β-D-glucuronide [141]. High plasma levels of pravastatin and repaglinide have been reported in subjects carrying the OATPC*5 allele [140], [141], [142], [143].

On the other hand, OATP1B1, OATP2B1, and OATP1B3 are mainly expressed on the hepatocyte sinusoidal membrane, which can facilitate the hepatic drug uptake [138]. OATP1B1 is encoded by *SLCO1B1* and is essential for the hepatic uptake of the simvastatin active metabolite, simvastatin acid [144]. Six important SNPs identified in the *SLCO1B1* gene with their allelic frequencies and functional consequences in Asian, African and Caucasian have been discussed in Table S3. Among them, the 521T > C variant of the *SLCO1B1* is associated with reduced OATP1B1 activity, which is responsible for the higher blood concentrations of simvastatin acid, as well as the consequently increased toxicity and reduced efficacy of simvastatin [145]. In addition, OATP1B1*15 was associated with increased plasma concentrations of pravastatin and 7-ethyl-10-hydroxycamptothecin (irinotecan active metabolite), whereas OATP1B1*17 variant is linked with an increased cholesterol synthesis mediated by pravastatin [146], [147], [148].

### OCTs

OCTs are proteins encoded by the *SLC22A* family and in humans, which are present in the basolateral cell membrane of the renal proximal tubule [149]. Three isoforms, *OCT1*, *OCT2*, and *OCT3*, have been identified in humans [150], [151], [152] and *OCT2* is highly expressed in the kidneys. OCTs mediate the cellular uptake of a wide range of structurally-different organic cations including clinically-administered drugs such as metformin and procainamide [150]. Metformin, a therapeutic agent used to treat type 2 diabetes mellitus, is predominantly renally excreted [153]. The *OCT2* 270S variant has been associated with low activity while the 270A variant induces high activity of OCT2 [153], [154]. Patients with type 2 diabetes who are homozygous for the 270A variant exhibit a significantly higher renal clearance and lower plasma concentration of metformin than those with the homozygous 270S variant [153], [154], [155]. On the other hand, allele variants G401S, R61C, G465R, and M420del are associated with lower OCT1 activities, which are responsible for the significantly increased renal clearance and reduced glucose-lowering effects of metformin in healthy subjects [156].

## Influence of genetic polymorphisms of drug metabolizing enzymes or transporters on drug–drug interactions

Effects of one drug are modified by other concomitantly administered drugs due to drug–drug interactions, which may be attributed to the altered pharmacokinetic or pharmacodynamic properties of one drug induced by the coadministered drug. The polymorphisms in drug metabolizing and transporter genes are an important risk factor of drug–drug interactions and varied interindividual drug responses [157]. These polymorphisms can lead to decreased levels of a drug-metabolizing enzyme in an individual, which may cause severe adverse drug reactions following the coadministration of enzyme inhibitors [158], [159]. Among the CYPs, CYP2C9, CYP2C19, and CYP2D6 are involved in the metabolism of approximately 40% of routinely administered drugs [160]. Different CYP allelic variants significantly contribute to the variability of an individual’s susceptibility to drug–drug interactions and drug-metabolizing capacities [161]. Different drugs interact with the CYP metabolic machinery differently. The metabolism of some drugs by CYP enzymes is extremely specific, for example, metoprolol is primarily metabolized by CYP2D6 [162], whereas other drugs such as warfarin may be simultaneously metabolized by several CYPs including CYP2D6, CYP3A4, and CYP1A2 [163]. Polymorphisms related to the altered expression of drug metabolizing and transporter genes will ultimately affect the therapeutic effects of administered drugs [73], [164]. When a drug is metabolized by more than one CYP metabolic pathway and the administered drug acts by inhibiting or inducing CYPs, genetic polymorphisms could redirect the metabolism of drugs via other CYP routes [162]. This could lead to drug–drug interactions. For example, antifungal voriconazole is actively metabolized by CYP3A4 and CYP2C19, whereas ritonavir strongly inhibits CYP3A4 while inducing CYP2C19 metabolic activities [165], [166]. When CYP2C19 PM patients are treated with voriconazole and ritonavir, up to 461% increased AUC of voriconazole was observed, since the patients were unable to metabolize voriconazole owing to reduced CYP2C19 and CYP3A4 activities [167], [168]. In another case, the antiplatelet activity of clopidogrel was reduced when it was administered with proton pump inhibitors such as esomeprazole and omeprazole owing to the inhibition of CYP2C19 [169], whereas an increased activity of clopidogrel was anticipated in the presence of rifampicin and aspirin [170]. Clopidogrel is a prodrug that needs oxidative activation *in vivo* by CYP1A2, CYP2B6 and CYP2C19 for its anti-platelet activity [171]. Genetic polymorphisms in *CYP2C19*, *CYP1A2*, 2B6*6, and CYP3A5*3 were found to be associated with the varied degree of drug–drug interactions for clopidogrel, due to its highly-complex pharmacokinetics and variable drug response as compare to other anti-platelet drugs [172], [173], [174], [175], [176].

Mutations in the drug transporter genes also contribute to drug–drug interactions and adverse drug reactions. HMGCR inhibitors such as atorvastatin, rosuvastatin, and pravastatin are actively transported by OATP1B1 and ABCG2 [147]. The concomitant administration of cyclosporine (a potent inhibitor of OATP1B1 and ABCG2) with statins like rosuvastatin and pitavastatin will result in higher plasma levels of statins, leading to rhabdomyolysis [177]. Digoxin is potently cleared by MDR1, therefore its coadministration with verapamil, clarithromycin, or talinolol that inhibits MDR1 transport activity leads to increased plasma levels due to decreased renal clearance of the drug [178], [179].

## Conclusions

The genetic variations of CYPs and transporters have been described in diverse populations. In this review, we review the different allelic variants that are responsible for altered drug activities in diverse geographic regions. Some populations exhibited extremely high frequencies of allele variants that are associated with several significant clinical consequences. Taking advantage of pharmacogenomics, researchers have assessed some specific genetic variants responsible for the particular drug responses of individuals.

Whole genome SNP profiling, haplotyping, multigene analysis, and gene expression studies by biochip or microarrays are all in place to study drug responses of individuals, which would aid in drug discovery, development, and individualized treatments. Given the common variability in drug responses among patients, the optimization of dosage regimen at the individual level is not an easy task. Comprehensive appreciation of the contributing factors associated with interindividual and interethnic differences in medication responses is a must for the development of precision medicine, and help health-care professionals in recommending the proper treatment with the best possible beneficial outcomes while preventing unwanted drug effects in the particular patients. The development of clinical practice strategies based on accurate genotype testing will facilitate the enhanced understanding of altered drug responses and drug–drug interactions. Furthermore, the development of more reliable biomarkers based on polymorphisms in genes responsible for the adverse events will hopefully create strategies for administering drugs based on the genotype and phenotype of patients, to minimize unwanted drug reactions.

## Competing interests

The authors have declared no competing interests.

## Figures and Tables

**Table 1:** *CYP2D6* genotype-based phenotype groups of individuals

**Phenotype**	**Genotype**	**Refs.**
PM	CYP2D6*3–*8, *11,*16, *18–*21, *38, *40, *42, *44, *56, *62	[39]
EM	CYP2D6*2, *17 x 2, *27, *35, *39, *48	[40]
IM	CYP2D6*10, *14, *17, *18, *36, *41, *47, *49–*51, *54, *55,*57	[41], [42]
UM	CYP2D6*2XN (N = 2, 3, 4, 5 or 13)	[39], [44], [52], [53]

*Note:* Classification is based on the metabolic capabilities of CYP2D6 enzyme on probe substrate (bufuralol, debrisoquine, sparteine, and dextromethorphan) among the sampled individuals in different populations. PM, poor metabolizer; IM, intermediate metabolizer; EM, extensive metabolizer; UM, ultra-rapid metabolizer.

**Table 2:** *CYP2C19* genetic polymorphisms with their clinical consequences

**Drug**	**Therapeutic class**	**CYP2C19 phenotype**	**Clinical significances**	**Refs.**
Lansoprazole, omeprazole	Gastroenterology	PM	Increased half-life leading to high cure rates in individuals with PM genotypes, which is reversed in EMs	[83], [84]
Diazepam	Psychiatry	PM	Extended sedative effect due to increased half-life in PMs	[81], [82], [83]
Clopidogrel	Cardiology	PM or IM	Increased threat of frequent stroke, stent thrombosis, and myocardial infarction in PMs due to reduced conversion of parent drug to active metabolite. Reduced antiplatelet activity associated with increased threat of bleeding disorder in CYP2C19*17 patients	[173], [174], [175], [176]

*Note*: PM, poor metabolizer; IM, intermediate metabolizer; EM, extensive metabolizer.

## Supplementary material

Supplementary Table S1 Major human polymorphic CYP genes and distribution frequencies of their important variant alleles in diverse ethnicities

Supplementary Table S2 Common allele variants of human polymorphic *CYP* genes

Supplementary Table S3 Common polymorphisms in human drug transporter genes and their allele frequencies
