# Corrigendum to “Pharmacogenomics of Drug Metabolizing Enzymes and Transporters: Relevance to Precision Medicine” [Genomics Proteomics Bioinformatics 14 (5) (2016) 298–313]

**DOI:** 10.1016/j.gpb.2018.04.001

**Published:** 2018-04-22

**Authors:** Shabbir Ahmed, Zhan Zhou, Jie Zhou, Shu-Qing Chen

**Affiliations:** 1 Department of Precision Medicine and Biopharmaceutics, College of Pharmaceutical Sciences, Zhejiang University, Hangzhou 310058, China; 2 International Center for Precision Medicine, Zhejiang California International NanoSystems Institute, Hangzhou 310058, China

The editors regret there was an error in Figure 1 published in Issue 5, 2016. In the figure, “Pharmacodynamics” should be corrected to “Pharmacokinetics”, and “Pharmacokinetics” should be corrected to “Pharmacodynamics”. The corrected Figure 1 is shown below. The editors would like to apologize for any inconvenience caused.

**Figure 1:**
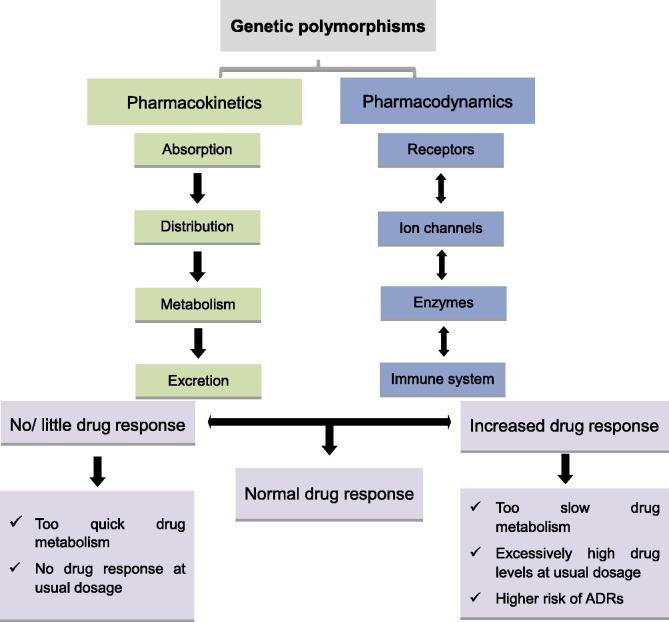
**Effect of genetic polymorphisms on individuals’ drug responses** Pharmacokinetics and pharmacodynamics are main determinants of interindividual differences in drug responses. Genetic polymorphisms in genes related to these processes may result in mild to severe variations in drug responses. ADRs, adverse drug reactions.

