# Supplementary material for: Pharmacogenomics of Drug Metabolizing Enzymes and Transporters: Relevance to Precision Medicine
Source: Genomics Proteomics Bioinformatics. 2016 Oct 8;14(5):298–313. doi: 10.1016/j.gpb.2016.03.008 (PMC5093856; doi:10.1016/j.gpb.2016.03.008)
Supplement: Supplementary Table S1 — Major human polymorphic CYP genes and distribution frequencies of their important variant alleles in diverse ethnicities [file mmc1.docx]

**Table S1 Major human polymorphic CYP genes and distribution frequencies of their important variant alleles in diverse ethnicities**

| **Gene** | **Population** | **No. of subjects** | **Frequency of important variant allele (%)** | | | | | | | | | | | **PMID** |
| --- | --- | --- | --- | --- | --- | --- | --- | --- | --- | --- | --- | --- | --- | --- |
| ***CYP2D6*** |  |  | ****1*** | ****2*** | ****2xN*** | ****3*** | ****4*** | ****5*** | ****6*** | ****10*** | ****14*** | ****17*** | ****41*** |  |
|  | Caucasians | 589 | 36.4 | 32.4 |  | 2.04 | 20.7 | 1.95 | 0.93 | 1.53 | 0 | 0 |  | 9012401 |
|  | Ethiopians | 122 |  |  | 16 | 0 | 1.2 | 3.3 |  | 8.6 |  | 9 |  | 8764380 |
|  | Chinese | 400 | 24.65 | 11.06 |  | 0 | 0.14 | 4.66 | 0 | 52.53 | 1.33 | 0 | 3.34 | 18632250 |
|  | Chinese-Hong Kong | 119 | 22.69 | 7.98 |  |  | 0 | 4.62 |  | 64.71 |  |  |  | 10620567 |
|  | African Americans | 154 | 34.7 | 26.9 |  | 0.3 | 7.8 | 6.2 |  | 7.5 |  | 14.6 |  | 11505219 |
|  | Ashkenazi Jewish | 250 | 28.6 |  |  |  | 22.6 |  |  |  |  |  |  | 18240905 |
|  | Basques | 76 | 40.79 | 32.89 | 0 | 0 | 21.05 | 2.63 | 1.32 | 1.32 |  | 0 |  | 15340360 |
|  | Canadian-Indians | 115 |  |  |  | 0 | 3 |  |  | 3 |  |  |  | 9170152 |
|  | Croatians | 200 | 76.5 | 4 |  | 2.75 | 14 | 1 | 1.5 |  |  |  |  | 12950145 |
|  | Faroeses | 309 | 61.6 |  |  | 0.2 | 33.4 |  | 1.9 |  |  |  |  | 16025294 |
|  | Gabonese | 154 | 32 | 44 |  |  |  |  |  |  |  | 24 |  | 10073750 |
|  | Germans |  |  |  | 1.8 |  | 21.3 | 2.1 |  | 1.6 |  |  |  | 9241658 |
|  | Greeks | 283 |  |  | 7.42 | 2.3 | 17.84 |  |  |  |  |  |  | 17635181 |
|  | Italians | 62 | 33.87 | 35.48 | 3.23 | 0 | 12.9 | 0 | 3.23 | 8.06 |  | 0 |  | 15340360 |
|  | Japanese | 206 | 43 | 12.3 |  |  | 0.2 | 4.5 |  | 38.1 | 0.7 |  |  | 10975611 |
|  | Jordanians | 192 |  |  |  |  | 12.8 |  |  | 14.8 |  | 8.3 |  | 22905959 |
|  | Koreans | 400 | 33.25 | 10.13 | 1.1 | 0 | 0.25 | 6.13 | 0 | 45 | 0.5 | 0 | 1.88 | 12089164  16778723 7550367  8946476 |
|  | Mexican Americans | 264 | 55.1 | 18 | 0.4 | 0.2 | 10 | 1.7 | 0.4 | 2.8 | 0 | 0.2 | 9.5 | 16283274 |
|  | Mexicans mestizo | 243 |  | 19.34 |  | 1.44 | 11.21 | 2.67 |  | 12.45 |  | 1.65 |  | 16249913 |
|  | Polish | 300 | 75.7 |  |  | 1.3 | 23 |  |  |  |  |  |  | 12536989 |
|  | Russian natives | 352 | 82.2 |  |  | 0.9 | 17 |  |  |  |  |  |  | 20373852 |
|  | Russians (European part) | 290 | 70.8 |  | 0.5 | 1 | 18.2 | 2.4 | 1.2 | 4.2 |  |  |  | 12879168 |
|  | Sardinians | 96 | 36.46 | 40.63 | 2.08 | 3.13 | 12.5 | 1.04 | 0 | 4.17 |  | 0 |  | 15340360 |
|  | Saudi Arabians | 192 |  |  | 10.4 |  | 3.5 | 1 |  | 3 |  | 3 | 18.4 | 24121619 9241658 |
|  | South Africans | 99 | 26.8 | 15.1 |  | 0 | 7.07 | 17.2 | 0 | 2.53 | 0.51 | 12.6 | 3.54 | 18202841 |
|  | South Indians | 447 | 46.1 | 34.8 |  | 0 | 7.3 | 1.9 |  | 10.2 | 0 | 0 |  | 16880622 |
|  | Spaniards | 102 | 41.18 | 35.29 | 0.98 | 0 | 17.65 | 1.96 | 0 | 0.98 |  | 0.98 |  | 15340360 |
|  | Subsaharan Africans | 1060 | 24.4 | 32.7 | 0.8 |  | 2.8 | 5.9 |  | 4.3 |  | 12.2 | 2.8 | 17301689 |
|  | Syrians | 102 |  | 47.06 | 3.92 | 0 | 9.8 | 0.98 | 0.98 | 2.94 |  | 0 |  | 15340360 |
|  | Tanzanians | 212 | 56.1 | 18.4 |  | 0.5 | 1.4 | 3.3 |  |  |  | 20.3 |  | 11372584 |
|  | Turkish | 404 | 37 | 35 | 6 |  | 11 | 1 |  | 6 |  | 1 |  | 10460072 17374963 |
|  | U.S. (Black) | 222 | 36–87 |  | 2.4 | 0.39–0.61 | 7.3–9.1 | 4.9–6.9 |  | 5.2 |  | 26 | 15 | 16550211 9918137 8098046 |
|  | Zimbabweans | 228 |  | 13 | 2 | 0 | 2 | 4 |  | 5.6 |  | 34 |  | 11372584  7908586 8971426 |
| ***CYP2C9*** |  |  | ****1*** | ****2*** | ****3*** |  |  |  |  |  |  |  |  |  |
|  | Caucasian | 111 | 95.5 | 10 | 7.4 |  |  |  |  |  |  |  |  | 14616425 |
|  | Chinese -Tibet | 96 | 93.75 | 0 | 5.73 |  |  |  |  |  |  |  |  | 25958051 |
|  | Bolivians | 778 | 92.2 | 4.8 | 3 |  |  |  |  |  |  |  |  | 15776277 |
|  | Koreans | 358 | 93.4 | 0 | 6 |  |  |  |  |  |  |  |  | 16187974 |
|  | Mexican | 98 | 86 | 8 | 6 |  |  |  |  |  |  |  |  | 15452553 |
|  | Cuban | 132 | 72 | 17 | 11 |  |  |  |  |  |  |  |  | 23959274 |
|  | Ecuadorian | 193 | 94 | 5.4 | 1.5 |  |  |  |  |  |  |  |  | 23171336 |
|  | African American | 600 | 86.7 | 2.8 | 2 |  |  |  |  |  |  |  |  | 20504253 |
|  | Malay | 209 | 95.7 | 1.9 | 2.4 |  |  |  |  |  |  |  |  | 19495518 |
|  | Vietnamese | 157 | 97.8 | 0 | 2.2 |  |  |  |  |  |  |  |  | 15795654 |
|  | Spanish | 1076 | 76.6 | 15.6 | 7.8 |  |  |  |  |  |  |  |  | 19381164 |
|  | Turkish | 499 | 79.4 | 10.6 | 10 |  |  |  |  |  |  |  |  | 10510154 |
|  | French | 151 | 77 | 15 | 8 |  |  |  |  |  |  |  |  | 12803577 |
|  | Portuguese | 135 | 78.8 | 13.2 | 8 |  |  |  |  |  |  |  |  | 18240903 |
|  | Ethiopians | 150 | 94 | 4 | 2 |  |  |  |  |  |  |  |  | 11678789 |
|  | Iranians | 152 | 64.88 | 25.34 | 9.8 |  |  |  |  |  |  |  |  | 20885015 |
|  | Pakistani | 74 | 73.6 | 12.1 | 14.1 |  |  |  |  |  |  |  |  | 25904339 |
|  | Chinese-Han | 2127 | 94.5 | 0.1 | 2.9 |  |  |  |  |  |  |  |  | 23400009 |
|  | Brazilian | 206 | 83.9 | 12.9 | 3.2 |  |  |  |  |  |  |  |  | 15660966 |
|  | Italians | 157 | 80 | 11 | 9 |  |  |  |  |  |  |  |  | 11678789 |
|  | Croatians | 200 | 74 | 16.5 | 9.5 |  |  |  |  |  |  |  |  | 12950145 |
|  | Egyptians | 247 | 82 | 12 | 6 |  |  |  |  |  |  |  |  | 12047484 |
|  | Faroeses | 311 | 86.7 | 8.8 | 5.3 |  |  |  |  |  |  |  |  | 16025294 |
|  | Greeks | 283 | 78.97 | 12.9 | 8.13 |  |  |  |  |  |  |  |  | 17635181 |
|  | Iranians | 400 | 87.25 | 12.75 | 0 |  |  |  |  |  |  |  |  | 17201743 |
|  | Ashkenazi Jewish | 250 | 77.2 | 14 | 8.6 |  |  |  |  |  |  |  |  | 18240905 |
|  | Japanese | 140 |  | 1 | 98.2 |  |  |  |  |  |  |  |  | 9631918 |
|  | Russians | 290 | 57.9 | 10.5 | 6.7 |  |  |  |  |  |  |  |  | 12879168 |
|  | South Africans | 152 | 78 | 22 | 0 |  |  |  |  |  |  |  |  | 11372584 |
|  | Tanzanians | 384 | 82 | 18 | 1.2 |  |  |  |  |  |  |  |  | 11372584 |
|  | Zimbabweans | 168 | 87 | 13 | 0 |  |  |  |  |  |  |  |  | 11372584 |
| ***CYP2C19*** |  |  | ****1*** | ****2*** | ****3*** |  |  |  |  |  |  |  |  |  |
|  | Italians | 360 |  | 11.1 | 0 |  |  |  |  |  |  |  |  | 15177309 |
|  | Iranians | 152 | 86.73 | 13 | 1 |  |  |  |  |  |  |  |  | 20885015 |
|  | African Americans | 236 | 81 | 18.2 | 0.8 |  |  |  |  |  |  |  |  | 16815315, 15499174 |
|  | Caucasians | 273 | 86.4 | 12.7 | 0.9 |  |  |  |  |  |  |  |  | 16815315 |
|  | Ashkenazi Jewish | 250 | 83 | 15.2 |  |  |  |  |  |  |  |  |  | 18240905 |
|  | Belgians | 121 | 90.9 | 9.1 | 0 |  |  |  |  |  |  |  |  | 14616425 |
|  | South Africans | 152 | 78 | 22 | 0 |  |  |  |  |  |  |  |  | 11372584 |
|  | Bolivians | 778 | 92.1 | 7.8 | 0.1 |  |  |  |  |  |  |  |  | 15776277 |
|  | Canadians (Indians) | 159 | 80.9 | 19.1 | 0 |  |  |  |  |  |  |  |  | 9797794 |
|  | Canadians (Inuit) | 152 | 89 | 11 | 0 |  |  |  |  |  |  |  |  | 8873219 |
|  | Greeks | 283 | 67.32 | 13.07 | 0 |  |  |  |  |  |  |  |  | 19102714 |
|  | Pakistani | 68 |  | 27.2 |  |  |  |  |  |  |  |  |  | 20102361 |
|  | Malaysians | 54 | 72 | 23 | 5 |  |  |  |  |  |  |  |  | 15327595 |
|  | Chinese | 400 | 69.73 | 24.67 | 3.27 |  |  |  |  |  |  |  |  | 18518848 |
|  | Chinese -Taiwanese | 118 | 63 | 32 | 5 |  |  |  |  |  |  |  |  | 9110363 |
|  | Chinese Han | 2127 | 61.42 | 33.07 | 5.34 |  |  |  |  |  |  |  |  | 23148634 |
|  | Burmeses | 127 | 66 | 30 | 4 |  |  |  |  |  |  |  |  | 16946555 |
|  | Colombian mestizo | 189 | 91.3 | 8.7 | 0 |  |  |  |  |  |  |  |  | 17623107 |
|  | Russians | 290 | 88.3 | 11.4 | 0.3 |  |  |  |  |  |  |  |  | 12879168 |
|  | Danish | 241 | 84 | 16 | 0 |  |  |  |  |  |  |  |  | 9754988 |
|  | Karens | 131 | 71 | 28 | 1 |  |  |  |  |  |  |  |  | 16946555 |
|  | Croatians | 200 | 85 | 15 | 0 |  |  |  |  |  |  |  |  | 12950145 |
|  | Egyptians | 247 | 88.8 | 11 | 0.2 |  |  |  |  |  |  |  |  | 12047484 |
|  | Tamil Indians | 112 | 60 | 38 | 2 |  |  |  |  |  |  |  |  | 12919183 |
|  | Ethiopians | 114 | 84 | 14 | 2 |  |  |  |  |  |  |  |  | 9014201 |
|  | European-Americans | 210 | 87 | 13 | 0 |  |  |  |  |  |  |  |  | 9110363 15499174 |
|  | Faroeses | 312 | 97.1 | 2.9 | 0 |  |  |  |  |  |  |  |  | 16025294 |
|  | Filipinos | 104 | 54 | 39 | 7 |  |  |  |  |  |  |  |  | 9110363 15499174 |
|  | Germans | 328 | 84 | 15.9 | 0.2 |  |  |  |  |  |  |  |  | 10460072 |
|  | Turkish | 404 | 88 | 12 | 0.4 |  |  |  |  |  |  |  |  | 10460072 |
|  | Zimbabweans | 168 | 87 | 13 | 0 |  |  |  |  |  |  |  |  | 11372584 |
|  | Iranians | 400 | 86 | 14 | 0 |  |  |  |  |  |  |  |  | 17201743 |
|  | Israeli Jewish | 140 | 84 | 15 | 1 |  |  |  |  |  |  |  |  | 10096259 |
|  | Koreans | 377 | 62 | 28 | 8 |  |  |  |  |  |  |  |  | 17667801 9797796 |
|  | Japanese | 253 | 60.1 | 26.7 | 12.8 |  |  |  |  |  |  |  |  | 16141610 |
|  | Tanzanians-Bantu | 251 | 81.5 | 17.9 | 0.6 |  |  |  |  |  |  |  |  | 9797796 |
|  | Mexican Americans | 346 | 90.2 | 9.7 | 0.1 |  |  |  |  |  |  |  |  | 16815315 |
|  | North Indians | 121 | 70 | 30 | 0 |  |  |  |  |  |  |  |  | 11014415 |
|  | South Indians | 341 | 64 | 35 | 1 |  |  |  |  |  |  |  |  | 15660966 |
|  | Northeastern Thai | 107 | 71 | 27 | 2 |  |  |  |  |  |  |  |  | 11927837 |
|  | Thai | 774 | 62 | 29 | 3 |  |  |  |  |  |  |  |  | 16946555 |
|  | Palestinians | 252 | 91.3 | 5.8 | 3 |  |  |  |  |  |  |  |  | 19193970 |
|  | Saudi Arabians | 194 | 85 | 15 | 0 |  |  |  |  |  |  |  |  | 9110363 15499174 |
|  | Vietnamese | 165 | 69 | 24 | 5 |  |  |  |  |  |  |  |  | 17667801 |
|  | Dutch | 765 | 86.5 | 13.3 | 0.2 |  |  |  |  |  |  |  |  | 11829201 |
|  | Swedish | 175 | 76.6 | 23.1 | 0.3 |  |  |  |  |  |  |  |  | 8747407 |
| ***CYP3A4*** |  |  | ****2*** | ****3*** | ****18*** |  |  |  |  |  |  |  |  |  |
|  | Caucasians | 426 | 2.7 | 1.1 | 0 |  |  |  |  |  |  |  |  | 11714865 11375299 |
|  | Chinese | 672 | 0 | 0 | 18 |  |  |  |  |  |  |  |  | 22224559 10668853 |
|  | Italians | 54 |  | 0.7 |  |  |  |  |  |  |  |  |  | 20931330 |
|  | Africans | 95 | 0 | 0 |  |  |  |  |  |  |  |  |  | 15882469 |
|  | Europeans | 95 | 0 | 2 |  |  |  |  |  |  |  |  |  | 15882469 |
|  | Koreans | 53 |  |  | 1.2 |  |  |  |  |  |  |  |  | 19020497 |
|  | Japanese | 118 |  |  | 1.3 |  |  |  |  |  |  |  |  | 15618745 |
|  | Chinese-Taiwan | 180 |  |  | 3.4 |  |  |  |  |  |  |  |  | 11714865 |
|  | Asians |  |  |  | 2 |  |  |  |  |  |  |  |  | 11714865 |
|  | Finnish | 118 | 2.7 |  |  |  |  |  |  |  |  |  |  | 10668853 7668294 |
| ***CYP3A5*** |  |  | ****2*** | ****3*** | ****6*** | ****7*** |  |  |  |  |  |  |  |  |
|  | Caucasians | 500 | 1 | 91.7 | 0.1 | 0 |  |  |  |  |  |  |  | 12324482 |
|  | Canadians | 160 | 0.7 | 92.9 | 0 | 0 |  |  |  |  |  |  |  | 11740341 |
|  | British | 100 |  | 94 | 0 | 0 |  |  |  |  |  |  |  | 12814460 |
|  | Zimbabweans | 200 | 0 | 77.6 | 22 | 10 |  |  |  |  |  |  |  | 15833928 |
|  | Italians | 54 | 0 | 93.3 | 0.7 |  |  |  |  |  |  |  |  | 20931330 |
|  | South Indians | 652 | 0 | 63.5 | 0 | 0 |  |  |  |  |  |  |  | 21265876 |
|  | Bulgarians | 146 | 0.6 | 93.1 | 0 |  |  |  |  |  |  |  |  | 17992026 |
|  | Asians | 220 |  | 75 | 0 | 0 |  |  |  |  |  |  |  | 11502729 |
|  | African-American | 90 | 0 | 27-50 | 13-16 | 10 |  |  |  |  |  |  |  | 11279519 |
|  | Jordanians | 591 | 0.2 | 86.6 | 1.7 | 0 |  |  |  |  |  |  |  | 22426036 |
|  | South African | 241 |  | 29.4 | 18.2 | 0.7 |  |  |  |  |  |  |  | 15978331 |
|  | Japanese | 265 | 0 | 74 | 0 |  |  |  |  |  |  |  |  | 12242601 |
|  | Chinese-Taiwanese | 180 |  | 69.2 |  |  |  |  |  |  |  |  |  | 16924387 |
|  | Greeks | 283 |  | 94.35 |  |  |  |  |  |  |  |  |  | 17635181 |
|  | Koreans | 2178 |  | 79.5 |  |  |  |  |  |  |  |  |  | 19020497 |
