# Supplementary material for: Pharmacogenomics of Drug Metabolizing Enzymes and Transporters: Relevance to Precision Medicine
Source: Genomics Proteomics Bioinformatics. 2016 Oct 8;14(5):298–313. doi: 10.1016/j.gpb.2016.03.008 (PMC5093856; doi:10.1016/j.gpb.2016.03.008)
Supplement: Supplementary Table S2 — Common allele variants of human polymorphic CYP genes [file mmc2.docx]

**Table S2 Common allele variants of human polymorphic *CYP* genes**

| **Gene** | **Chr** | **Variant allele** | **Polymorphism** | **Functional effect on enzyme activity** | **Allelic frequency (%)** | **Population** | **Clinical consequence** | **PMID** |
| --- | --- | --- | --- | --- | --- | --- | --- | --- |
| ***CYP2D6*** | 22 | **2xN* | Gene duplication  /multiduplication | Increased enzyme expression/amount  resulting in UM | 10−30 | Saudi Arabians and Africans | Highly-reduced plasma drug levels resulting in the loss of drug efficacy with higher drug dose required; increased ADRs due to increased metabolite or active drug production (ADRs after treatment with codeine) | 8764380 9241658 |
|  |  | **3* | Frameshift mutation | Inactive enzyme  resulting in PM | 3.13 | Sardinians | High plasma drug level due to reduced metabolism | 15340360 9012401 |
|  |  | **4* | Defective splicing | Inactive enzyme  resulting in PM | 23−33 | Polish and Faroeses | Reduced drug dose recommended | 16025294 12536989 |
|  |  | **5* | Gene deletion | Abolished enzyme activity resulting in PM | 5.9−6.2 | Spaniards and African Americans | Increased risk of drug-related adverse effects | 17301689 11505219 |
|  |  | **6* | Frameshift mutation | Inactive enzyme  resulting in PM | 1.9−3.3 | Faroese and Italians | Higher levels of the parent drug (*e.g.*, tamoxifen), increased risk for ADRs | 16025294 |
|  |  | **10* | P34S, S486T | Unstable enzyme  resulting in reduced enzyme activity and IM | 53−65 | Chinese | Lower dose for some patients, decreased active metabolite formation (tamoxifen) | 18632250 17470523 |
|  |  | **17* | T107I, R296C, S486T | Reduced enzyme activity due to altered substrate affinity, resulting in IM | ~30 | Africans | Increased concentration of parent drug, lower drug dose recommended | 11372584 17470523 |
|  |  | **41* | Splicing defect | Inactive enzyme  resulting in IM | 18.4 | Saudi Arabians | Lower dose required for some patients. Over all *CYP2D6* polymorphisms affect pharmacokinetics and response of many drugs, including tricyclic neuroleptics, analgesics, antidepressants, antiarrhythmics, antiemetics, and anticancer drugs | 24121619 |
| ***CYP2C9*** | 10 | **2* | R144C | Decreased enzyme activity due to reduced affinity for CYP oxidoreductase | ~25 | Iranians | Affecting the oral clearance of substrate drugs like ibuprofen, phenytoin, celecoxib, and S-warfarin | 20885015 |
|  |  | **3* | I359L | Reduces enzyme activity due to altered substrate specificity | 14 | Pakistanis |  | 25904339 |
| ***CYP2C19*** | 10 | **2* | Defective splicing | Inactive enzyme  resulting in PM | 35 | South Indians | Affecting the pharmacokinetics and/or response of several drug classes, including proton pump inhibitors (*e.g.*, omeprazole) and barbiturates | 15660966 |
|  |  | **3* | W212X, I331V/ premature stop codon | Inactive enzyme  resulting in PM | ~13 | Japanese |  | 16141610 |
| ***CYP3A4*** | 7 | **2* | S222P | Decreased enzyme activity | ~3 | Caucasians | Substrate-dependent altered enzyme activity | 11375299 |
|  |  | **3* | M445T | Decreased catalytic activity | 2 | Europeans | Minor or moderate clinical relevance. No consensus on direct functional or clinical association | 15882469 |
|  |  | **18* | L293P | Increased enzyme activity | 18 | Chinese |  | 10668853 |
| ***CYP3A5*** | 7 | **2* | T398N |  | 1 | Dutch Caucasians | Associated with the pharmacokinetics of the immunosuppressive drug tacrolimus | 11740341 |
|  |  | **3* | Defective splicing | Abolished enzyme expression and catalytic activity | > 94 | Greeks |  | 17635181 |
|  |  | **6* | Defective splicing | Inactive enzyme | 22 | Zimbabweans |  | 15833928 |
|  |  | **7* | Frameshift mutation | Severely-reduced enzyme activity | 10 | African-Americans |  | 11279519 |

*Note:* Chr, chromosome; ADR, adverse drug reaction; PM, poor metabolizer; IM, intermediate metabolizer; EM, extensive metabolizer; UM, and ultra-rapid metabolizer.
