# Supplementary material for: Pharmacogenomics of Drug Metabolizing Enzymes and Transporters: Relevance to Precision Medicine
Source: Genomics Proteomics Bioinformatics. 2016 Oct 8;14(5):298–313. doi: 10.1016/j.gpb.2016.03.008 (PMC5093856; doi:10.1016/j.gpb.2016.03.008)
Supplement: Supplementary Table S3 — Common polymorphisms in human drug transporter genes and their allele frequencies [file mmc3.docx]

**Table S3 Common polymorphisms in human drug transporter genes and their allele frequencies**

| **Class** | **Gene** | **Important variant allele (mutation)** | **Frequency (%)** | | | **Functional consequence** | **PMID** |
| --- | --- | --- | --- | --- | --- | --- | --- |
|  |  |  | **Asian** | **African** | **Caucasian** |  |  |
| ***ABC*** | *ABCB1* | 1236 C>T (silent) | 60–72 | 15–21 | 34–42 | Affecting co-translational folding in nearby amino acids that are essential for ATP binding and hydrolysis | 19285158 14749689 |
|  |  | 3435C>T (silent) | 37–66 | 10–27 | 48–59 | Affecting co-translational folding of amino acid residues nearby, thereby altering substrate specificity | 19285158 17185560 |
|  |  | *ABCB1*13* (1236C>T/2677G>T  /3435C>T haplotype) | 28–56 | 4.5–8.7 | 23–42 | Affecting the inhibition by a small subset of modulators | 17700595 |
|  |  | 2677G>A (A893T) | 3–22 |  | 1–10 | Affecting gene expression or protein function (inconsistent data) | 18287207 |
|  | *ABCC1* | 128G>C (C43S) | 1 |  |  | Reduced plasma membrane localization and decreased vincristine resistance in transfected cells | 21929509  12731862 |
|  |  | 1299G>T (R433S) |  |  |  | Changed transport and resistance | 12042670 |
|  |  | 2012G>T (G671V) |  | 1.4 | 2.8 | Associated with anthracycline-induced cardiotoxicity | 15709111 16330681 |
|  | *ABCC2* | 1271A>G (R412G) |  |  |  | Associated with reduced methotrexate elimination in DJS patients | 12942343 15864128 15821043 |
|  |  | 1249G>A (V417I) | 13–19 | 14 | 22–26 | Changed protein expression and localization; associated with anthracycline-induced cardiotoxicity. | 24743544 |
|  |  | 3563T>A (V1188E) | 1 |  | 4–7 | Associated with anthracycline-induced cardiotoxicity | 15709111 |
|  |  | 4544G>A (C1515Y) |  |  | 4–9 | Associated with anthracycline-induced cardiotoxicity | 15709111 |
|  | *ABCG2* | 34G>A (V12M) | 15–18 | 4–6 | 2–10 | Altered transport properties and resistance | 17297656 16608919 |
|  |  | 376C>T (Q126stop) | 0.9–1.7 | 0 | 0 | Loss of transport activity | 14750175 |
|  |  | 421C>A (Q141K) | 27–35 | 1–5 | 9–14 | Affecting the ATP-binding domain and leading to decreased transport activity | 16608919 |
| *OATP* | *SLCO1A2 (OATP1A2)* | 38T>C (I3T) | 0 | 2.1 | 11.1 | Increased transport activity | 19290786 |
|  |  | 516A>C (E172D) | 0 | 2.1 | 5.3 | Associated with decreased transport activity | 19290786 |
|  |  | 833A (N278del) | 0 | 0.6 | 0 | Decreased transport activity | 19290786 |
|  | *SLCO1B1 (OATP1B1)* | 217T> C (F73L) | 0 | 0 | 2 | Decreased transport activity | 16525793 |
|  |  | 388A>G (N130D) | 54 | 74 | 30 | Decreased transport activity | 16525793 |
|  |  | 463C>A (P155T) | 0 | 2 | 16 | No effect on transport activity. | 16525793 |
|  |  | 521T>C (V174A) | 0.7 | 2 | 14 | Decreased transport activity | 16525793 |
|  |  | 1463G>C (G488A) |  | 9 | 0 | Decreased transport activity | 16525793 |
|  |  | 2000A>G (E667G) | 0 | 34 | 2 | Decreased transport activity | 16525793 |
|  | *SLCO1B3 (OATP1B3)* | 334T>G (S112A) |  |  | 74 | Unknown effect | 16525793 |
|  |  | 699G>A (M233I) |  |  | 71 | Unknown effect | 16525793 |
|  |  | 1564G>T (G522C) |  |  | 19 | Affecting localization, resulting in reduced transport activity | 16525793 |
|  | *SLCO2B1 (OATP2B1)* | 1457C>T S486F | 30.9 |  | 1.2 | Decreased transport activity | 16525793 |
| *OCT* | *SLC22A1 (OCT1)* | 41C>T (S14F) | 0 | 3.1 | 0 | Involved in reduced metformin transport but increased transport of MPP | 16504381 |
|  |  | 480C>G (G160L) | 8.6–13.0 | 0.5 | 0.65 | No effect on transport activity | 19290786 |
|  |  | 1022C>T (P341L) | 16 | 8.2 | 0 | Decreased transport of MPP but not metformin | 22345987 |
|  |  | 1201G>A (G401S) | 0 | 0.7 | 1.1 | Decreased transport activity | 25398212 |
|  |  | 1222A>G(M408V) | 74–81 | 74 | 60 | No effect on transport activity | 18466105 |
|  |  | 1256delATG(M420del) | 0 | 2.9 | 18 | Decreased transport of metformin but not MPP | 18762711 |
|  | *SLC22A2 (OCT2)* | 596C>T (T199I) | 1 | 0 | 0 | Decreased transport activity | 18762711 |
|  |  | 602C>T (T201M) | 1.3–2.0 | 0 | 0 | Decreased transport activity | 18466105 |
|  |  | 808GVT (A270S) | 14–17 | 11 | 16 | Decreased transport activity | 16164626 |
|  |  | 1198C>T (R400C) | 0 | 1.5 | 0 | Decreased transport activity | 23691375 |
|  |  | 1294A>C (K432Q) | 0 | 1 | 0 | Decreased transport activity | 9325277 |
| *OAT* | *SLC22A6 (OAT1)* | 20T>C (L7P) | <1 | 1 | 1 | Reduced protein expression | 17397052 |
|  |  | 149G>A (R50H) | 1 | 1 | 1 | Increased transport activity | 15102542 |
|  |  | 1361G>A (R454Q) | 0 | <1 | 0 | Decreased transport activity | 16164626 |
|  | *SLC22A8 (OAT3)* | 523A>G (I175V) | 1 | 1 | 1 | Unknown effect | 16164626 |
|  |  | 829C>T (R277W) |  |  |  | Decreased transport activity | 17103332 |

*Note:* ABC, ATP-binding cassette; DJS, Dubin-Johnson Syndrome; OCT, organic cation transporter; OAT, organic anion transporter; OATP, organic anion transporting polypeptide; MPP, 1-methyl-4-phenylpyridinium.
